# What are the current indications for use of radiofrequency devices in hip arthroscopy? A systematic review

**DOI:** 10.1093/jhps/hnv055

**Published:** 2015-08-11

**Authors:** Carlos Suarez-Ahedo, S. Pavan Vemula, Christine E. Stake, Zachary A. Finley, Timothy J. Martin, Chengcheng Gui, Benjamin G. Domb

**Affiliations:** 1. American Hip Institute, Westmont, IL, USA; 2. Hinsdale Orthopaedics, Hinsdale, IL, USA; 3. Loyola University Chicago Stritch School of Medicine, Maywood, IL, USA

## Abstract

The role of radiofrequency energy (RFE) devices has been minimally studied in hip arthroscopy. The purpose of this study was to determine the role of RFE devices in hip arthroscopy through a systematic review of the literature. We searched the PubMed database using the following Medical Subject Heading terms: hip arthroscopy, hip radiofrequency, thermal capsulorrhaphy, thermal chondroplasty and radiofrequency debridement. Two authors independently reviewed the literature and included articles based on predetermined inclusion criteria. We excluded review, technique and experimental articles. After title and abstract review, we selected 293 articles for full-text review. Ten articles met the inclusion and exclusion criteria. For the included articles, a total of 305 hips underwent arthroscopy with concomitant RFE treatment at a mean age of 25.7 years. Eight articles presented patient-reported outcome (PRO) instruments, one study did not report an outcome instrument but utilized an evaluation of postoperative range of motion (ROM) and 1 year magnetic resonance image (MRI) and computed tomography (CT) imaging. The remaining article measured only the ROM pre- and postoperatively. Only one of the articles reviewed reported complications. Current evidence on the safety and indications for use of RFE devices in hip arthroscopy is insufficient. The literature shows mixed results regarding its use in hip arthroscopy. Although the use of thermal energy is not without risk, if used judiciously and appropriate precautions are taken to avoid damage to adjacent tissues, those devices can be useful for the treatment of certain intra-articular hip pathologies arthroscopically.

## INTRODUCTION

Several tools such as radiofrequency energy (RFE) devices have been developed for the thermal modification of soft tissue structures [1]. RFE devices generate energy resulting in heat, in accordance with Joules Law, on tissue adjacent to the device due to molecular friction caused by the oscillation of intracellular and extracellular ions [2, 3]. Since the end of nineteenth century, multiple studies were conducted on the use of RFE for the treatment of medical conditions such as malignant lesions of the skin, cervix, oral cavity and bladder [2].

 For adequate function, RFE devices require the formation of a closed-loop electrical circuit consisting of a radiofrequency generator in conjunction with the patient and properly placed electrodes in order to complete the electrical circuit [2]. Monopolar RFE devices include one active electrode and one dispersive electrode, which is generally a grounding pad on the patient. In bipolar devices the energy current passes and is concentrated between the electrodes located within the surgical instrument [3]. The resulting heat at high temperatures (100°C) causes the destruction of collagen molecules, resulting in adjacent tissue ablation. However, when the temperature is in a range between 40 and 70°C, denaturation of the collagen triple helix is obtained which results in shortening the length of the structure [4]. Another effect observed with the use of this type of energy is the sealing effect, resulting in a decrease of water permeability of the tissue (specifically cartilage), keeping its morphology with compressive forces [5].

The clinical usefulness of RFE in arthroscopic surgery has been extensively investigated for the treatment of joint capsular laxity, synovitis, chondral lesions and soft tissue injury. Although there is an abundance of literature regarding the use of RFE devices in the shoulder and in the knee, the results are varied and inconclusive in nature. Further still, the development of clinical studies comparing the use and results of those devices in the hip joint are limited. The primary challenge of RFE in hip arthroscopy is the anatomy of the joint requires the development of special designs of RFE devices [3].

Primary concerns regarding the use of thermal energy in arthroscopic surgery of the hip are risks of irreversible cartilage damage, osteonecrosis, potential progression of the lesion treated with energy, damage of surrounding tissues and finally the risk of thermal burn with the use of these devices. This warrants a better understanding of the safety of RFE devices.

The purpose of this study was to determine what are the current indications and efficacy of the RFE devices for the treatment of pathologies of the hip arthroscopically by systematically reviewing the literature. A secondary objective was to report the safety for the use of RFE devices in hip arthroscopy.

## METHODS

In January 2015, we searched the PubMed database using the following key words: hip arthroscopy, hip radiofrequency, thermal capsulorrhaphy, thermal chondroplasty, radiofrequency debridement. Two independent reviewers (CSA and SPV) reviewed the titles and abstracts to select relevant articles for full-text review. Both reviewers then examined the full-text articles for eligibility. Articles were included based on the following criteria: (i) were in the English language, (ii) studies composed of all levels of evidence, (iii) performed on human patients and (iv) studied the utility and/or safety using the RFE devices in hip arthroscopy. We excluded review articles, technique articles, articles with overlapping patient populations, experimental articles, and studies using an animal model. Additionally, the bibliographies of identified articles were searched for relevant articles for full-text review.

We then performed a full-text review of the included articles to determine the patient demographics, clinical presentation, diagnosis, mean follow-up, results and complications related with the use of RFE devices.

## RESULTS

In January 2015, our literature search identified 1714 articles from PubMed database. After title and abstract review, we selected 292 articles for full-text review. Of these, 10 articles met the inclusion criteria for this systematic review. The characteristics and findings of the remaining articles that have been selected for this systematic review are presented in Table I.

**Table I:** Findings from selected articles

Study	Year	Level of evidence	Number of hips	Mean age in years	Follow-up in months (range)	PROI	Tissue treated	Results	Complications
Más Martínez *et al*.	2015	Case Report	1	58	24 months	mHHS	Acetabular cartilage		Femoral head chondrolysis//hip resurfacing
Amenabar *et al*.	2013		27 with isolated partial thickness, Type 2 LT tears	24.4	32 (23–49)	mHHS	LT and capsule	17.2% of the patients treated without LT debridementand capsular tighthening had recurrence of their symptoms	Not reported
						NAHS			
								mHHS score improves 24.1 points with RF LT debridement and RF capsulorrhaphy (*P* < 0.05)	Not reported
								NAHS score improves 20.5 points with RF LT debridement and RF capsulorrhaphy. (*P* < 0.05)	
								One patient developed recurrent symptoms requiring second hip arthroscopy finding synovitis in the superior capsule, away from the area of previous capsular RF treatment, and there was no LT abnormality.	Not reported
Lee *et al*.	2013		2	55.5	24 and 36, respectively	HHS	Paralabral Cysts	HSS improved by 35 points in the first case and 46 points in the second case	Not reported
						WOMAC		WOMAC improved by 43 points in the first case and 45 points in the second case	
						UCLA activity score		UCLA activity scores improved 4 points in the first case and 5 points in the second case	
Ricci *et al*.	2013		1	47	22	None	Osteoid osteoma	At the end of follow-up the patient had complete ROM without pain	Not reported
								MRI and CT at 1 year follow-up showed no pathologic signs or synovitis.	
Polesello *et al*.	2013	IV	9	35	32 (22–45)	mHHS	GM Tendon	The mean modified HHS increased (*P* = 0.011) from 61.3 ± 7.5 points (range, 45–70 points) preoperatively to 77.6 ± 11.9 points (range, 62–93 points) at latest follow-up.	Not reported
Philippon MJ.	2001		12	31	12	mHHS	Capsule	83% had significant improvement in their symptoms	Not reported
Yu-Jie Liu *et al*.	2009	IV	216	23.7	17.4 (7–42)	None	GMC	Adduction (10.4° ± 7.2°) and flexion (44.8° ± 14.1°) before surgery increased after surgery atfinal follow-up adduction (45.3° ± 8.7°) and flexion (110.2° ± 11.9°)(both *P* < 0.001)	Not reported
Ilizaliturri *et al*.	2009	I	19	29.5 (group 1) and 36.2 (group 2)	12	WOMAC	Iliopsoas Tendon	Improvements in WOMAC scores were statistically significant in both groups (group 1: *P* = .0001; group 2: *P* = .001)	Not reported
Ilizaliturri *et al*.	2006	IV	11	26	24	WOMAC	Iliotibial band	WOMAC improved by 13 points with 100% resolution of pain and 91% resolution of snapping symptoms	Not reported
Ilizaliturri *et al*.	2005	IV	7	38.5	21.4 (10–27)	WOMAC	Iliopsoas Tendon	patients improved an average of 8.5 points on the WOMAC scale	Not reported

There were four articles designated as Level IV evidence and one article reporting Level I evidence. There were a total of 305 hips that underwent an arthroscopic procedure with concomitant RFE treatment, with a mean age of 25.7 years.

### Iliopsoas release

Ilizaliturri *et al*. [6, 7] reported in two articles the use of an RFE device to release the iliopsoas tendon arthroscopically. In the first article there were a total of 26 hips, which demonstrated improvement on the WOMAC scales, without any complications related with the use of the RFE devices. In this study, they compared iliopsoas release using RFE at different levels and presented no controls.

### Chondroplasty

Más Martínez *et al*. [8] recently published a case report of a patient who underwent hip arthroscopy for treatment of a chondral lesion on the acetabular side for which microfracture was used concomitantly with RFE. Two months later the patient’s mHHS scores improved from 39.6 to 69.3 postoperatively. Four months after the initial surgery the patient starts with pain in the same hip, requiring a hip resurfacing, they reported a chondral lesion on the femoral head of a patient, which, they believed was caused by the use of RFE in the first hip arthroscopy. However, there is no conclusive evidence in their assertion. The other nine articles did not report complications regarding the usage of RFE devices.

### Tenotomy

Polesello *et al*. [9] described the use of the thermal energy to tenotomize the Gluteus Maximus tendon near its insertion at the linea aspera for external snapping hip syndrome in nine hips, at minimum 22 months follow-up, average mHHS was 61 points preoperatively and 78 points at latest follow-up and no neurovascular complications were reported. Ilizaliturri *et al*. described the use of RFE devices to release the iliotibial band in patients diagnosed with external snapping hip syndrome; their results show an improvement of 13 points in the WOMAC scale with 100% resolution of pain and 91% resolution of snapping symptoms [10]. Liu *et al*. [11] described the use of thermal energy to release the contractures of the gluteal muscle as due to their use of benzyl alcohol in 108 patients (216 hips). The author reported an improvement of 34.9 degrees in adduction and 65.4 degrees in flexion, both statistically significant with a *P* < 0.001.

### Ligamentum debridement

One article by Amenabar *et al*. [12] identified 27 hips with isolated partial thickness type 2 ligamentum teres tears out of 1574 patients, average age was 24.4 years, which were treated with partial debridement using an RFE device. In those cases the capsule tightening was performed by thermal shrinkage in 24 hips and three hips were treated by suture plication. Over an average follow-up of 32 months the authors demonstrated an significant improvement on the preoperative mHHS and NAHS < (0.05) in the RFE group. No complications were reported by the use of thermal energy.

### Capsule

Philippon *et al*. [13] evaluated the use of thermal energy to treat patients with hip instability. The study included 12 pts, 11 of which reported outcomes (mHHS increased 69 to 87 at 6 months of follow-up) for patients exhibiting symptoms of traumatic and idiopathic hip instability.

### Cystic lesions

Lee *et al*. [14] report the use of RFE devices in hip arthroscopy, as their method to treat a paralabral cysts in two patients. In their first case, a 70-year-old female, with final follow-up 2 years postoperatively, the HSS improved from 58 to 93 points, WOMAC score improved from 69 to 26 points, and the UCLA activity score improved from 4 to 8 points. Their second case, a 41-year-old female, the patient returned to full participation in activities of daily living, 3 months after the surgery. At final follow-up 3 years postoperatively, the HHS improved from 52 to 98 points, the WOMAC score improved from 61 to 16 points, and the UCLA activity score improved from 4 to 9 points. Otherwise, Ricci *et al*. [15] described the use of RFE to treat an osteoid osteoma in the hip. At 22-month follow-up, the patient had no pain, a normal gait and normal ROM.

## DISCUSSION

This review of the use of RFE in hip arthroscopy is the first systematic review to address the indications and the safety of use of TE to treat intra or extra-articular pathologies of the hip. We reviewed ten articles showing different indications for the use of RFE specifically in hip arthroscopy for multiple pathologies.

Although the use of this technology contains certain risks, only one of the articles reviewed showed complications after the use of TE in hip arthroscopy. Based on our findings, this suggests that these devices can be used for tenotomies, and ligamentum debridement in an effective fashion. These indications for RFE in the hip were the most comprehensively studied of any the uses. Furthermore they presented the most patients and best outcomes. However, while there were studies suggesting that chondroplasty and capsule shrinkage could be treated by RFE, the literature is scarce and the outcomes are inconclusive.

Traditionally, the treatment to chondral damage represents a challenge to the surgeon due to its limited ability for spontaneous regeneration, friability and the difficult to replace properties of biological hyaline cartilage. It is for these reasons that there are multiple operative procedures for its treatment including Autologous Chondrocyte Implantations (ACI), Mosaicplasty, Osteochondral Allograft Transplantation and Articular Cartilage Repair including mechanical shaving and Thermal Chondroplasty. RFE is indicated in patients with focal cartilage lesions to stabilize the chondral lesion, sealing fibrillation and creating a plasma layer in a conductive fluid resulting in better cartilage smoothness and stiffness. With the use of mechanical shaver, iatrogenic damage can be caused to surrounding cartilage. The temperature that RFE devices can reach can cause a detrimental effect on chondrocyte viability [16]. Based on this knowledge, Lu *et al*. [17] performed a controlled laboratory study to compare the effect on a chondromalacic samples using a monopolar thermal chondroplasty in a 37°C lavage solution versus a 22°C lavage solution. Using confocal laser microscopy and electron microscopy they found that thermal chondroplasty with 37°C lavage solution resulted in less depth of chondrocyte death and produced smoother surfaces than with 22°C solution for 10 s of treatment. However, bipolar and monopolar devices delivers energy in different manners, including how they measure the energy that they release [18–20].

Barber *et al*. [21] reported in a randomized trial the treatment of grade III chondral lesions using a monopolar RFE and compared the effects on subchondral bone with a MRI 12 months after surgery, whereupon no evidence of heat-related subchondral damage such as avascular necrosis (AVN) was found. Voloshin *et al*. [22] treated 25 chondral lesions using bipolar RFE device, and during the second look arthroscopy, more than 50% showed partial or complete filling of the defect. Although reported complications regarding the use of RFE in knee arthroscopy are few, the quality of clinical evidence about safety and efficacy remains low, our suggestion is to be cautious and judicious using this technology until future research has clearly defined the long-term clinical outcomes and risks.

Spahn *et al*. [23] compared clinical results after bipolar RFE chondroplasty to mechanical debridement. The authors found that the group of patients treated using a bipolar RFE device returned to activity significantly earlier than the group treated with mechanical debridement (*P* = 0.002). Additionally, for patients in the RFE device treated group, visual analog scale (VAS) pain scores were significantly lower (*P* < 0.001), demonstrated significantly improved Tegner scores (*P* < 0.001) and Knee injury and Osteoarthritis Outcome Score (KOOS) domain scores for pain, symptoms, activities of daily living (ADL) and Quality of Life (QOL) (*P* < 0.001).

Uthamanthil *et al*. [24] compared the use of monopolar, bipolar and mechanical debridement in an animal model. They found that permeability on the cartilage treated with bipolar RFE devices decreased, inferring that this may lead to longer-term stability to the cartilage. Otherwise, the amount of thermal exposure may prove to be a critical factor. Edwards *et al*. [19] looked at the effect on human cartilage of six RFE treatment time intervals 5, 10, 15, 20, 30 and 40 s in six independent samples (36 treatments/RFE generator). The depth of chondrocyte death was measured using confocal laser microscopy (CLM) and scanning electron microscopy (SEM). Authors concluded that bipolar RFE produced significantly greater chondrocyte death than monopolar RFE at identical treatment time intervals, which can be theorized because less energy is required by the bipolar RFE devices to produce the same effect. However, it took at least 15 s for bipolar and monopolar RFE to smooth a 1-cm^2^ grade 2 chondromalacic lesion as determined by SEM.

Hecht *et al*. [25] evaluated the thermal effect of monopolar RFE on the mechanical, morphologic and biochemical properties of joint capsule in an *in vivo* animal model. They concluded that the use of thermal energy initially caused a detrimental effect on the mechanical properties of the capsule, associated with partial denaturation of the tissue. This was followed by gradual improvement of the mechanical, morphologic and biochemical properties of the tissue over time.

Thermal capsulorrhaphy to treat joint instability has been used since its introduction in 1994. The thermal energy is applied to capsular tissue to shrink and thicken, reducing the redundancy. Usually, the temperature is set between 70 and 80°C, without exceeding 100°C. The shrinkage is caused by thermal denaturation of collagen (a major component of joint capsular tissue) [26].

Favorito *et al*. [27] investigated the effect of arthroscopic laser-assisted capsular shift in patients with multidirectional shoulder instability. The study reported an overall success rate of 81.5%. Selecky *et al*. [28] and Ferrara *et al*. [29] investigated the effect of Ho:YAG laser energy and monopolar RFE, respectively, on the biomechanical properties of the inferior glenohumeral ligament complex using shoulders from cadavers. Those studies showed that the strength of the ligament complex was not altered significantly. Both authors concluded that the specimen was able to sustain a greater amount of stretch before failure and the strength of the inferior glenohumeral ligament complex was not significantly compromised.

There are reports in the literature regarding thermal skin burns in patients’ undergoing hip arthroscopy. Curtin *et al*. [30] presented a 29 year-old female who 3 days after hip arthroscopy presented with an erythematous and tender area between the two portal sites, but improved at 6 weeks. Kouk *et al*. [31] reported a case in a 53-year-old male who had a second-degree burn of the shoulder and chest wall as a result of fluid overheating due to RFE. Both authors recommend raising awareness of the effect of elevated irrigation fluid temperatures on the patient’s skin.

Further studies are needed to analyse the long-term results and effects of RFE energy in hip arthroscopy. Due to the lack of literature, and several obfuscating variables, controlled studies need to be undertaken to evaluate the usage of RFE versus non-RFE treatment of pathology in the hip to see if it may be of clinical relevance for hip arthroscopy with an aim to improve patient outcomes.

## LIMITATIONS

Limitations in our systematic review include the small number of articles discussing the use of thermal energy in the arthroscopic hip literature. We elected to exclude articles including animal models or ex vivo models. In having an environment that can be controlled, several situations that can arise during surgery are eliminated and thus alter the results. Furthermore, the effectiveness of RFE during hip arthroscopy was not measured through uniform PRO outcomes. Various scores were used to evaluate patients, limiting our ability to compare studies Also, RFE was used for different indications across these studies, and thus it is difficult to evaluate optimal usage of RFE technology.

## CONCLUSIONS

Current evidence on the safety and indications for use of RFE devices in hip arthroscopy is insufficient. The literature shows mixed results regarding its use, as mainly studies have focused on various uses of RFE in the hip and have presented case studies or smaller populations. Although the use of thermal energy is not without risk, if it is used judiciously and appropriate precautions to avoid damage to adjacent tissues, those devices may be useful for the treatment of certain intra-articular hip pathologies arthroscopically and needs to be evaluated further.

## CONFLICT OF INTEREST STATEMENT

Dr. Domb reports personal fees and other from Arthrex, INC, personal fees and other from Stryker MAKO Surgical Corp, other from Breg, other from ATI, personal fees and other from Pacira, personal fees from Orthomerica, personal fees from DJO Global, personal fees from Amplitude, outside the submitted work; and Dr. Domb is a boardmember for American Hip Institute, which funds research and is the institute where our studies are performed. Dr. Domb is also a boardmember at the AANA Learning Center Committee.
